# Clinical Significance of Non-Invasive Prenatal Screening for Trisomy 7: Cohort Study and Literature Review

**DOI:** 10.3390/genes12010011

**Published:** 2020-12-24

**Authors:** Xiaofan Zhu, Doris Yuk Man Lam, Matthew Hoi Kin Chau, Shuwen Xue, Peng Dai, Ganye Zhao, Ye Cao, Sunny Wai Hung Cheung, Yvonne Ka Yin Kwok, Kwong Wai Choy, Xiangdong Kong, Tak Yeung Leung

**Affiliations:** 1 Genetics and Prenatal Diagnosis Center, Department of Obstetrics and Gynaecology, The First Affiliated Hospital of Zhengzhou University, Zhengzhou 450052, China; zhuxf@link.cuhk.edu.hk (X.Z.); rarfen@163.com (P.D.); zhaoganye@163.com (G.Z.); 2 Department of Obstetrics and Gynaecology, The Chinese University of Hong Kong, Hong Kong, China; matthewchau@link.cuhk.edu.hk (M.H.K.C.); xuesw@link.cuhk.edu.hk (S.X.); yecao@cuhk.edu.hk (Y.C.); kky254@ha.org.hk (Y.K.Y.K.); richardchoy@cuhk.edu.hk (K.W.C.); 3 NIPT Department, NGS Lab, Xcelom Limited, Hong Kong, China; doris.lam@xcelom.com (D.Y.M.L.); sunny.cheung@xcelom.com (S.W.H.C.); 4 Shenzhen Research Institute, The Chinese University of Hong Kong, Shenzhen 518100, China; 5 The Chinese University of Hong Kong-Baylor College of Medicine Joint Center for Medical Genetics, Hong Kong, China

**Keywords:** non-invasive prenatal screening, cell-free DNA screening, cell-free fetal DNA, trisomy 7, uniparental disomy 7

## Abstract

Trisomy 7 is the most frequently observed type of rare autosomal trisomies in genome-wide non-invasive prenatal screening (NIPS). Currently, the clinical significance of trisomy 7 NIPS-positive results is still unknown. We reviewed two independent cohorts from two laboratories where similar NIPS metrics were applied. A total of 70,441 singleton cases who underwent genome-wide NIPS were analyzed, among which 39 pregnancies were positive for trisomy 7, yielding a screen-positive rate of 0.055% (39/70,441). There were 28 cases with invasive testing results available; the positive predictive value (PPV) was 3.6% (1/28). We then searched the published NIPS studies to generate a large cohort of 437,873 pregnancies and identified 247 cases (0.056%) that were screened positive for trisomy 7. The overall PPV was 3.4% (4/118) in the combined data. The presence of uniparental disomy 7 was not detected in the NIPS trisomy 7-positive pregnancies with normal fetal karyotype. Among the 85 cases with pregnancy outcome available in combined data, 88.2% were normal live births, 14.1% had intrauterine growth restriction, preterm birth or low birth weight, 3.5% presented with ultrasound abnormality, and no fetal loss was observed. Our data provide valuable information for counseling and management of trisomy 7-positive NIPS pregnancies.

## 1. Introduction

Chromosomal abnormalities affect one of 150 live births, and the prevalence is even higher in pregnancies [1,2]. Prenatal diagnosis of chromosomal abnormalities can be performed by using advanced technologies such as chromosomal microarray analysis (CMA) and genome sequencing [3,4], which, however, requires an invasive procedure such as amniocentesis or chorionic villi sampling (CVS) to obtain a fetal sample for testing. These procedures carry a 0.1–0.2% procedure-related miscarriage risk [5]. Fetal trophoblastic cells isolated from maternal blood allow for the reliable detection of fetal aneuploidies and copy number variants (down to 1 to 2 Mb in size) without maternal DNA contamination, but several limitations still prevent the clinical application of this cell-based method, such as that the risk of too few cells recovered and the specific isolation of fetal cells from the maternal circulation [6]. In addition, there are very few studies about detecting rare autosomal trisomies using fetal cells present in maternal blood [7]. Recently, cell-free DNA (cfDNA) in maternal circulation, which is released by placenta trophoblast cells as well as DNA in the extracellular vesicles (evDNA) [8,9], is widely used for noninvasive prenatal screening (NIPS, or non-invasive prenatal testing) for common aneuploidies in the clinical setting.

By use of massively parallel sequencing, cfDNA fragments from whole-genome are sequenced in NIPS, but only common trisomies are analyzed and reported. Before genome-wide NIPS was introduced into clinical use, genome-wide chromosomal abnormalities, including the rare autosomal trisomies (RATs), were under-investigated, even though large quantities of sequencing data from other chromosomes are also generated. Currently, more than 10 studies have reported the observations of RATs detected through genome-wide NIPS [10]. The clinical consequences of RATs are highly variable, depending on the specific chromosomes involved, the proportion of trisomic cells in the embryonic and extraembryonic lineages, and the presence of uniparental disomy (UPD) resulting from a trisomic rescue.

Trisomy 7 was the most frequently observed type of RATs in genome-wide NIPS, accounting for 23% of the 775 RATs cases reported previously [10,11,12,13]. The frequency was strikingly similar to that reported in the direct analysis of chorionic villi sampling (CVS) [10]. This suggests that cfDNA and direct preparation of CVS are originating from the same cell lineage. Pregnancies with trisomy 7 are thought to result in spontaneous miscarriage at early gestation, but through trisomic rescue, one copy of the extra chromosome 7 may be lost, and UPD or biparental disomy (BPD) will arise [14]. Silver-Russell syndrome is associated with maternal UPD7 in approximately 10% of cases; chromosomal mosaicism, in combination with UPD, was also found in prenatal cases [15].

Interestingly, trisomy 7 identified in the CVS analysis was never confirmed by amniocentesis, suggesting the trisomic cells are most likely confined to the placenta [10,14,16]. In a recent genome-wide NIPS study by Qi Y et al., where 35 cases with positive trisomy 7 NIPS results were retrospectively reviewed [17], trisomy 7 was not confirmed in any cases who received the diagnostic testing by amniocentesis. Further investigations on 10 cases where the placenta was available indicated all these trisomy 7 NIPS-positive samples were placental mosaicism for chromosome 7 [17]. However, in other studies, there was true fetal mosaicism of trisomy 7 confirmed in cases with NIPS-positive for trisomy 7, although complete clinical follow-up was still lacking [18,19]. Herein, we investigated the trisomy 7 screened positive cases based on genome-wide NIPS data from two independent clinical laboratories and reviewed the significance of NIPS-positive findings of trisomy 7 in published studies.

## 2. Materials and Methods

### 2.1. Study Subjects

We performed a retrospective review of all the singleton pregnancies who were referred for genome-wide NIPS in two independent clinical laboratories using similar NIPS quality control metrics and analytical pipelines. Cohort 1 (CUHK) included data from 39,134 singleton pregnancies tested by genome-wide NIPS in The Chinese University of Hong Kong (SafeT21express) between 2015 and 2018. Cohort 2 (ZZU) consisted of 31,307 singleton pregnancies from the Genetics and Prenatal Diagnosis Center in The First Affiliated Hospital of Zhengzhou University between 2018 and 2019. Pretest and post-test counseling were provided by obstetricians, written informed consents were obtained from all the pregnant women for the storage and genetic analysis of prenatal and maternal samples. Cases detected with isolated trisomy 7 findings were included, and 3 cases with dual aneuploidies were excluded for analysis, including trisomies 7 and 8, trisomies 7 and x, and trisomies 7 and monosomy 15.

### 2.2. Genome-Wide NIPS

Ten milliliters of maternal peripheral blood were collected into a Streck BCT tube and proceeded for genome-wide NIPS by using the protocols as previously reported [20]. All procedures and molecular tests, including cell-free DNA isolation, library construction, sequencing and bioinformatics analyses, were performed at the two clinical laboratories. Following library construction and amplification, the samples were sequenced on the Nextseq500 (Illumina, San Diego, CA, USA) with a minimum of 15–20 million read pairs per sample in cohort 1 and cohort 2. The scope of detection and reporting of the genome-wide NIPS test included risk assessment for common trisomies and genome-wide chromosomal aberrations at a resolution of 3Mb or above. In cohort 1, combined count-based and size-based analyses were conducted for the detection of chromosomal aberrations [13,21], and the count-based method was performed in cohort 2.

### 2.3. Invasive Diagnostic Testing and Clinical Follow-Up

For the cases with trisomy 7 identified by genome-wide NIPS, invasive prenatal diagnostic procedure and confirmation testing of G-banded karyotyping and chromosomal microarray analysis (CMA) were performed. For those who received a normal karyotype result, the SNP array test was further conducted to examine the presence of UPD. Pregnancy outcome information was obtained from referring doctors.

### 2.4. Summary of Published Data

A literature search by using the PubMed database was conducted to identify genome-wide NIPS studies that have reported the detection of trisomy 7. The searches were using the keywords “non-invasive prenatal testing (NIPT)” or “cell-free DNA”, or “genome-wide NIPT” or “expanded NIPT” and “rare autosomal trisomy”. The inclusion criteria for study selection was that trisomy 7 was described in the positive RATs results of expanded NIPS, and the case number was available. Proof-of-concept studies, case reports or other papers where cases were biasedly selected were excluded from the analysis.

## 3. Results

### 3.1. Characteristics of Pregnancies Positive for Trisomy 7 by NIPS

Between 2015 and 2018, a total of 39,134 consecutive singleton cases from cohort 1 were referred for genome-wide NIPS. In cohort 2, a total of 31,307 singleton cases have undergone genome-wide NIPS between 2018 and 2019. Collectively, there were 23 and 16 singleton pregnancies screened positive for trisomy 7 alone in cohort 1 and cohort 2, yielding a screen-positive rate of 0.059% (23/39,134) and 0.051% (16/31,307), respectively. Characteristics of these screen-positive cases within each cohort are shown in Table 1. The median maternal age at sampling was 32 and 30 years, and gestational age was 14.3 and 18.4 weeks in cohort 1 and cohort 2, respectively. The median maternal body mass index (BMI) was 20.2 kg/m^2^ and 23.0 kg/m^2^, there were 13% (3/23) and 18.8% (3/16) cases with a BMI above 25 kg/m^2^ in cohort 1 and cohort 2, respectively (Table 1). Among the 39 positive cases, 22 were females, and 17 were males.

### 3.2. Confirmatory Diagnostic Testing and Clinical Follow-Up

Invasive prenatal confirmation results on amniotic fluid (AF) were available for 14 cases in cohort 1. Among these, mosaic trisomy 7 was detected in one case; all the remaining 13 cases had a normal karyotype (Table 2). Thus, the positive predictive value (PPV) of trisomy 7-positive NIPS result was estimated to be 7.1% (1/14). Karyotyping of placental tissues was performed for 3 cases, among which two cases had a normal result (Table 2). In the other case (15H04940), which was confirmed as true fetal mosaicism, non-mosaic trisomy 7 was detected in the placenta. SNP-array analysis on the AF samples of 11 normal euploid fetuses also excluded the presence of UPD7 (Table 2). In addition, pregnancy outcome was available in 8 cases, among which 6 were live birth without gross malformations. The true positive case underwent termination of the pregnancy without gross anomalies observed in a postmortem examination. The remaining case ended with preterm birth, and the birth weight was low (2.36 kg); subsequent development was observed to be normal until 3 years old (Table 2).

Of the 16 screen-positive cases in cohort 2, 14 received invasive prenatal diagnostic testing, and the results were normal by karyotype. The other 2 subjects refused invasive confirmation testing. Follow-up studies showed all the 16 pregnancies ended with live birth and developmental milestones were normal by the time of the interview, except one with ventricle septal defect detected by ultrasound.

### 3.3. Published Data Analysis

Collectively, 13 studies were included for analysis of trisomy 7 detected by genome-wide NIPS. The frequency of trisomy 7 varied more than 17-fold among different studies, ranging from 0.014% to 0.25% (Table 3). In the combined data with ours, the overall observed rate of positive results for trisomy 7 in genome-wide NIPS was 0.056% (1/1772). Despite the incomplete follow-up, the majority of cases were not confirmed in the fetuses who have undergone invasive confirmatory tests by amniocentesis or cord blood. In total, only 4 cases were confirmed in the fetuses, while 114 were not confirmed by amniocentesis or cord blood test, with an estimate of PPV of 3.4% (4/118). Interestingly, all of these 4 cases were true fetal mosaicism. UPD testing was performed in 3 studies, but no UPD7 was identified, consistent with our results (Table 3).

In addition, clinical follow-up information was documented in 61 cases across 8 studies. Among the 61 cases, the most common pregnancy outcome was live birth, involving 53/61 (86.9%). After pooling with our data, a total of 85 cases have clinical follow-up available, among which 88.2% (75/85) pregnancies ended with live birth (Table 3). Fetal growth restriction or preterm birth, low birth weight was reported in 12 of 85 cases, and ultrasound abnormality was observed in 3 (of 85) cases. No fetal loss was observed in all the 85 cases.

## 4. Discussion

In this study, we retrospectively reviewed 39 cases screened positive for trisomy 7 among the data retrieved from two independent clinical laboratories. The frequency was similar in cohort 1 and cohort 2 (0.059% vs. 0.051%). By contrast, the frequency in published data varied by more than 17-fold, ranging from 0.014% (~1 in 7237) [13] to 0.25% (1/399) [24]. This could be due to the limited sample size studied. For the first time, we reported the PPV of trisomy 7-positive NIPS to result in a large cohort of pregnancies (*n* > 400,000) was as low as 3.4%, despite follow-up was not complete. Although the overall PPV in our study was comparable to the pooled PPV, there was a significant difference in PPV in cohort 1 (7.1%) and 2 (0%). Notably, no UPD7 was identified in pregnancies with normal fetal karyotype both in our study and previous reports, suggesting that trisomic zygote rescue rarely occurs in these fetuses. Overall, the pregnancy outcome was satisfactory, except there were a minority of cases with FGR, preterm birth and low birth weight. No fetal loss was observed in these pregnancies.

Non-mosaic trisomy 7 is thought to result in spontaneous miscarriage at early gestation; it has not been described in liveborn infants in our study. When encountering the positive result of trisomy 7 by expanded NIPS in the clinical setting, it is speculated that the trisomy 7-positive finding is more likely to be present in mosaic form, either confined to the placenta or fetal mosaicism. The similarity of the chromosome distributions of RATs detected by NIPS and CVS analysis has indicated that fetal cfDNA and direct CVS preparation are from the same cell lineage [10]. In addition, the majority of these cases have normal karyotypes by AF analysis. Thus, it is possible that the trisomy 7 detected by genome-wide NIPS is mainly attributed to CPM (confined placenta mosaicism). This may explain the low PPV for trisomy 7-positive results by genome-wide NIPS in our study, as the data also suggested that confirmation by amniocentesis is recommended in clinical practice.

Although the combined frequency (0.056%) in our study was comparable to the previous review [10], there was large heterogeneity of the frequencies estimated among different expanded NIPS studies; this could be owing to the use of different criteria and detection algorithms. A deeper sequencing depth is more likely to identify the low-level mosaicism and yield a positive test result. Therefore, a clinical validation study is needed for evaluating the performance of various test platforms. Nevertheless, a placental study was not always conducted in previous expanded NIPS studies; there were very few positive cases studied and confirmed in the placenta. The study by Qi Y et al. included placenta examination in 8 of 29 cases with trisomy 7 alone; this is the largest case number of placenta studies for trisomy 7 screened positive cases up to date [17]. In our study, we also examined the placenta in three (of 14) cases in cohort 1; non-mosaic trisomy 7 in the placenta was identified in one case, which was confirmed with true fetal mosaicism by AF analysis. However, we could not exclude the possibility of sample selection bias, as placenta biopsy is a random selection process, the karyotype in the selected sites may not be the same as the ones of other sites in the placenta. In addition, the mosaic level also varies in different CPM cases [14]. Therefore, CPM7 cannot be eliminated in the other two cases with normal placental karyotype. Biopsy of multiple sites of the placenta is warranted when examining the presence of CPM7 in future studies.

As chromosome 7 is a well-known imprinting chromosome, it is very crucial to examine the presence of UPD7 in the fetus when encountering trisomy 7 in the pregnancy. Our SNP-based CMA results revealed that most of the CPM7 cases were hypothesized to be the result of somatic duplication events within the placental lineage rather than trisomic zygote rescue; therefore, the risk of fetal UPD7 is low [14]. In an early study by Kalousek et al., only a single fetus with matUPD was identified among the CPM7 cases, indicating the meiotic origin of CPM, while in the other 8 eight cases, biparental inheritance was demonstrated [14]. Recently, Benn P et al. showed that none of 93 CPM7 cases were confirmed with UPD7 detected in the AF sample in their study [10]. In the combined data of our study, we showed no UPD7 cases had been documented in the confirmation tests. Our results support that normal fetal karyotype in the trisomy 7 NIPS positive cases is less likely to result from a trisomic rescue.

Different from trisomy 7, confined placental mosaicism involving other chromosomes such as trisomy 16 and trisomy 22 may have clinical implications for the early prediction of adverse pregnancy outcomes. For example, the presence of CPM16 is well-known to be associated with adverse outcomes, such as preeclampsia, fetal growth restriction and prematurity [29]. Therefore, whether screening for genome-wide RATs should be provided in the clinical service is still on debate [30].

In recent years, NIPS has been increasingly offered as a public-funded service for women with a positive result by traditional screening in order to reduce the demand for subsequent invasive testing [11,12,31,32]. Professional guidelines have recommended cfDNA screening for common aneuploidies as a primary test offered to all pregnant women [33,34]. Considering the low PPV of trisomy 7, as well as the relatively high discordance rate between NIPS and invasive testing (CMA) for rare aneuploidies, expanded or genome-wide NIPS should not be recommended for pregnant women, especially for high-risk pregnancies [35]. This echoes the statement in the professional guidelines [33,34]. However, for women at increased risk of chromosomal abnormalities but desire a noninvasive screening approach, expanded NIPS can be an alternative, but appropriate pre- and post-test counseling should be performed regarding the limitation of the test as well as the meaning of a positive or negative result. For pregnancies with trisomy 7-positive NIPS result but receiving a negative invasive diagnostic result, close monitoring should also be provided as ultrasound anomalies and pregnancy complications can occur in more than 10% of pregnancies.

## 5. Conclusions

In this study, we reported for the first time the PPV for trisomy 7-positive results in a large dataset of genome-wide NIPS; this information is very useful for genetic counseling regarding the likelihood of carrying an affected fetus for NIPS trisomy 7-positive pregnancies. Considering the high possibility of CPM, an amniocentesis will be more informative than CVS at an early gestation age. Further investigations on the placenta are needed to clarify the biological reason for the positive finding. Although very rare, fetal UPD7 should also be taken into consideration when encountering CPM7. Furthermore, a clinical validation study is warranted to investigate the technical issues and CPM contributing to the positive trisomy 7 signal. Since there usually are no obvious anomalies detected in trisomy 7 screen-positive cases and the PPV for a positive trisomy 7 result is low, expanded or genome-wide NIPS for screening trisomy 7 will cause maternal anxiety and increase the demand for invasive procedures. In addition, the pregnancy outcome is overall satisfactory for these cases, but pregnancy complications such as fetal growth restriction can still occur. Therefore, it will be challenging for genetic counseling and pregnancy management of NIPS trisomy 7-positive cases.

## Figures and Tables

**Table 1:** Characteristics of the 39 cases screened positive for trisomy 7 in cohort 1 and cohort 2.

	Cohort 1 (*n* = 23)	Cohort 2 (*n* = 16)	Cohort 1 vs. Cohort 2 (*p* Value)
Maternal age (years)			
Median (range)	32 (23–48)	30 (23–40)	0.217
Mean ± SD	33.1 ± 6.3	30.9 ± 4.7	
Maternal BMI (kg/m^2^)			
Median (range)	20.15 (16.61–27.04)	23.04 (18.13–26.17)	<0.05
Mean ± SD	20.90 ± 2.59	22.6 ± 2.5	
GA at sampling (weeks)			
Median (range)	14.3 (10–37.4)	18.4 (12–32.3)	<0.05
Mean ± SD	15.6 ± 5.6	19.3 ± 4.6	
NIPT result			
Fetal fraction (%)			
Median (range)	15.91 (5.4–30.79)	15.1 (10–34.6)	0.899
Mean ± SD	15.99 ± 5.55	17.24 ± 7.21	
Count-based Z score			
Median (range)	23.9 (9.25–53.8)	6.7 (3.08–21.79)	<0.05
Mean ± SD	24.2 ± 10.9	8.8 ± 5.7	
Size-based Z score			
Median (range)	14.1 (5.27–36.6)	NA	
Mean ± SD	16.2 ± 7.7	NA	

**Table 2:** Invasive prenatal diagnosis and pregnancy outcome of 28 cases with trisomy 7-positive NIPS findings. CMA, chromosomal microarray analysis; UPD, uniparental disomy; AF, amniotic fluid; NA, not available; TOP, termination of pregnancy; NLB, normal live birth; LBW, low birth weight; VSD, ventricle septal defect.

Cohort	Sample No.	Specimen Type	Karyotyping	CMA	UPD7 Study	Pregnancy Outcome
1	15H01341	AF	46,XX	Normal	Negative	NA
	15H04940	AF	47,XX,+7 [9]/46,XX[21]	arr[GRCh37]7p22.3q36.3(203558-158928217) × 2~3	Negative	TOP, no gross anomaly, vitals stable
		Placenta	47,XX,+7	NA	NA	
	15H05302	AF	46,XX	NA	NA	NLB
		Placenta	46,XX	NA		
	15H05415	AF	46,XY	arr[GRCh37]2p11.2p11.1(89488303-91803693) × 3 pat, 11p13(31532618-31723695) × 1 mat	Negative	NLB
	16H02801	AF	46,XY	Normal	Negative	NLB
	16H03054	AF	46,XX	Normal	Negative	NLB
	16H12580	AF	46,XY	NA	Negative	NA
	16H14290	AF	46,XX	Normal	Negative	NA
	17H03630	AF	46,XY	Normal	Negative	Preterm birth, LBW
	17H04911	AF	46,XY	Normal	Negative	NLB
		Placenta	46,XY	NA		
	17H08559	AF	46,XX	Normal	Negative	NLB
	17H11388	AF	46,XY	NA	Negative	NA
	18H11643	AF	46,XX	Normal	NA	NA
	18H15500	AF	46,XX	NA	NA	NA
2	P18ZD0463	AF	NA	Normal	NA	NLB
	P190143	AF	NA	Normal	NA	NLB
	P190179	AF	NA	Normal	NA	NLB
	P190403	AF	NA	Normal	NA	NLB
	P19E0424	AF	NA	Normal	NA	NLB
	P19E1600	AF	NA	Normal	NA	NLB
	P19E2037	AF	NA	Normal	NA	NLB
	P192187	AF	NA	Normal	NA	NLB
	P192113	AF	NA	Normal	NA	NLB
	P19E2978	AF	NA	Normal	NA	NLB
	P19E3421	AF	NA	Normal	NA	NLB
	P194278	AF	NA	Normal	NA	NLB
	P194963	AF	NA	Normal	NA	VSD, NLB
	P19A0044	AF	NA	Normal	NA	NLB

**Table 3:** Observed trisomy 7-positive cases in genome-wide cell-free DNA (cfDNA) analysis in the publications. CPM, confined placental mosaicism; UPD7, uniparental disomy 7; USG, ultrasonography; TOP, termination of pregnancy; IUGR, intrauterine growth restriction; PB, preterm birth; LBW, low birth weight; LB, live birth.

Study	Trisomy 7 (n)	Total Cases (n)	Prevalence (%)	Invasive Confirmation				Clinical Follow-Up				
				Confirmed *	Not Confirmed ^#^	CPM7	UPD7	n	USG Anomaly	TOP	IUGR, or PB, LBW	LB
Brady, P. (2016) [22]	3	4000	0.075	0	2	-	-	-	-	-	-	-
Enrich, M. (2017) [23]	11	10,272	0.11	-	-	-	-	-	-	-	-	-
Fiorentino, F. (2017) [19]	4	12,114	0.033	1	3	-	-	4	-	-	0	4
Pertile, M.D. (2017) [18]	67	89,817	0.075	1	1	1	Negative (*n* = 1)	6	-	-	1	5
Pescia, G. (2017) [24]	16	6388	0.25	0	6	-	Negative (*n* = 6)	-	-		-	-
Scott, F. (2018) [25]	6	23,388	0.026	0	5	1	-	6	1	1	2	5
Van Opstal, D. (2018) [26]	6	2527	0.24	0	6	3	Negative (*n* = 3)	6	1	-	1	6
Wan, J. (2018) [27]	18	15,362	0.12	1	10 ^∆^	-	-	7	-	-	-	7
Chatron, N. (2019) [28]	1	1617	0.062	0	1	-	-	1	-	-	-	1
Liang, D. (2019) [13]	13	94,085	0.014	-	-	-	-	-	-	-	-	-
van der Meij, K.R.M. (2019) [11]	32	73,239	0.044	0	32	-	-	-	-	-	-	-
de Wergifosse, S. (2019) [12]	2	3373	0.059	0	2	-	-	2	-	-	1	2
Qi, Y. [17]	29	31,250	0.093	0	19 ^$^	8	-	29	-	-	6	23
Cohort 1 (CUHK)	23	39,134	0.059	1	13	1	Negative (*n* = 11)	8	-	1	1	6
Cohort 2 (ZZU)	16	31,307	0.051	0	14	-	-	16	1	-	-	16
**Total**	**247**	**437,873**	**0.056**	**4**	**114**	**14**	**Negative (*n* = 21)**	**85**	**3**	**2**	**12**	**75**

* Confirmed in the fetuses by amniocentesis or cord blood; ^#^ not confirmed means the trisomy 7 is not confirmed in the fetuses by amniocentesis or cord blood; ^∆^ included a positive CMA result: 7q11.23q21.11 × 2 hmz, 5.08 Mb; ^$^ included a positive CMA result: 7q11.23 (72,600,482-74,175,485) × 3.

## Data Availability

The raw datasets analyzed during the current study are not publicly available because the public access was not consented from the subjects in the study, but are available from the corresponding authors on reasonable request.
